# A_2A_ adenosine receptor upregulation correlates with disease activity in patients with systemic lupus erythematosus

**DOI:** 10.1186/s13075-016-1089-8

**Published:** 2016-08-26

**Authors:** Alessandra Bortoluzzi, Fabrizio Vincenzi, Marcello Govoni, Melissa Padovan, Annalisa Ravani, Pier Andrea Borea, Katia Varani

**Affiliations:** 1 Department of Medical Science, Section of Rheumatology, University of Ferrara, Ferrara and Azienda Ospedaliero-Universitaria Sant’Anna di Ferrara, Ferrara Cona (Ferrara) Via Aldo Moro 8 44124 Cona, Ferrara, Italy; 2 Department of Medical Sciences, Institute of Pharmacology, University of Ferrara, Via Fossato di Mortara 17-19, Ferrara, Italy

**Keywords:** Systemic lupus erythematosus, A_2A_ adenosine receptor, Disease activity

## Abstract

**Background:**

Adenosine is a purine nucleoside implicated in the regulation of the innate and adaptive immune systems, acting through its interaction with four cell surface receptors: A_1_, A_2A_, A_2B_, and A_3_. There is intense interest in understanding how adenosine functions in health and during disease, but surprisingly little is known about the actual role of adenosine-mediated mechanisms in systemic lupus erythematosus (SLE). With this background, the aim of the present study was to test the hypothesis that dysregulation of A_1_, A_2A_, A_2B_, and A_3_ adenosine receptors (ARs) in lymphocytes of patients with SLE may be involved in the pathogenesis of the disease and to examine the correlations between the status of the ARs and the clinical parameters of SLE.

**Methods:**

ARs were analyzed by performing saturation-binding assays, as well as messenger RNA and Western blot analysis, with lymphocytes of patients with SLE in comparison with healthy subjects. We tested the effect of A_2A_AR agonists in the nuclear factor kB (NF-kB) pathway and on the release of interferon (IFN)-α; tumor necrosis factor (TNF)-α; and interleukin (IL)-2, IL-6, IL-1β, and IL-10.

**Results:**

In lymphocytes obtained from 80 patients with SLE, A_2A_ARs were upregulated compared with those of 80 age-matched healthy control subjects, while A_1_, A_2B_, and A_3_ ARs were unchanged. A_2A_AR density was inversely correlated with Systemic Lupus Erythematosus Disease Activity Index 2000 score disease activity through time evaluated according to disease course patterns, serositis, hypocomplementemia, and anti-double-stranded DNA positivity. A_2A_AR activation inhibited the NF-kB activation pathway and diminished inflammatory cytokines (IFN-α, TNF-α, IL-2, IL-6, IL-1β), but it potentiated the release of anti-inflammatory IL-10.

**Conclusions:**

These data suggest the involvement of A_2A_ARs in the complex pathogenetic network of SLE, acting as a modulator of the inflammatory process. It could represent a compensatory pathway to better counteract disease activity. A_2A_AR activation significantly reduced the release of proinflammatory cytokines while enhancing those with anti-inflammatory activity, suggesting a potential translational use of A_2A_AR agonists in SLE pharmacological treatment.

## Background

Systemic lupus erythematosus (SLE) is the prototypic multisystem autoimmune disorder with a broad spectrum of clinical presentations encompassing almost all organs and tissues [1]. The irreversible break in immunological tolerance is manifested by immune responses against endogenous nuclear antigens and the subsequent formation of autoantibodies and immune complexes (ICs). SLE has classically been considered an autoimmune disease with a predominant adaptive immune system component because T and B cells have been considered the most important pathogenetic players [2].

During the early inflammatory phase, plasmacytoid dendritic cells (DCs) are able to internalize nucleic acids containing interferogenic ICs that reach the endosomes and stimulate Toll-like receptor 7 or 9, leading to interferon (IFN)-α gene transcription [3–5]. IFN-α contributes to the maturation of myeloid DCs that can activate autoreactive T cells through antigen presentation and costimulation. This favors the development of T helper 1 cells responsible for the high-level production of proinflammatory cytokines [6–8] and enhances B-cell maturation and differentiation, antibody production, and IC formation. In SLE, the IC- and IFN-α-secreting monocytes modulate interleukin (IL)-10 function [8]. The capability of IL-10 to suppress production of inflammatory cytokines such as tumor necrosis factor (TNF)-α and IL-6, implicated in promoting autoimmunity and tissue inflammation in SLE, is attenuated [8].

Growing evidence emphasizes that the purine nucleoside adenosine plays an active role as a local regulator of inflammation in different pathologies. Adenosine is a ubiquitous nucleoside involved in various physiological and pathological functions by stimulating the G protein-coupled A_1_, A_2A_, A_2B_, and A_3_ adenosine receptors (ARs) [9–12]. The role of ARs is well known in physiological conditions and in a variety of pathologies, including inflammatory damage, neurodegenerative disorders, and cancer [13–15]. In particular, A_2A_AR stimulation mediates inhibition of TNF-α, IL-1β, IL-2, IL-6, and IFN-α [16–18] and increases the production of the anti-inflammatory cytokine IL-10 [19]. With this background, the aim of the present study was to explore the arrangement and functionality of ARs in SLE and to evaluate their relationship with clinical phenotype and disease activity.

## Methods

### Patients and study design

Patients with SLE regularly attending our lupus clinic and satisfying the 1997 revised American College of Rheumatology criteria [20] were consecutively recruited from the Rheumatology Unit, Sant’Anna Hospital, University of Ferrara, Ferrara, Italy. We recorded clinical, demographic, and serological data, as well as data regarding therapy, including corticosteroids (measured as prednisone equivalent), antimalarials, and immunosuppressants.

Disease activity routinely assessed using the Systemic Lupus Erythematosus Disease Activity Index 2000 (SLEDAI-2 K) [21] and cumulative damage assessed with the Systemic Lupus International Collaborating Clinics Index were extracted by retrieving information from clinical records and a dedicated database. Moreover, disease activity and progress through time were considered according to four different patterns defined using the SLEDAI-2 K, excluding serological descriptors (hypocomplementemia and anti-double-stranded DNA [anti-dsDNA] antibodies) to focus on clinical activity: chronic active disease (CAD), relapsing-remitting disease, clinical quiescent disease (CQD), and minimal disease activity [22].

Seroimmunologic tests included complement components C3 and C4 dosages, antinuclear antibody (ANA), anti-dsDNA, anti-Sjögren’s-syndrome-related antigen A (anti-SSA), anti-Sjögren’s-syndrome-related antigen B (anti-SSB), anti-Smith (anti-Sm), antiribonucleoprotein (anti-RNP), anticardiolipin (aCL), anti-β_2_-glycoprotein I (anti-β_2_-GPI), and lupus anticoagulant (LA). C3 and C4 (in grams per liter) were measured by nephelometry, and hypocomplementemia was defined by local laboratory reference values (e.g., C3 < 0.8 g/L and C4 < 0.11 g/L detected on at least two separate occasions). ANA were detected by indirect immunofluorescence using HEp-2 cells as a substrate; positivity was defined as a titer ≥1:160. Anti-dsDNA were detected by indirect immunofluorescence using *Crithidia luciliae* with a cutoff titer of 1:40; positivity was certified if confirmed in two separate measurements. Anti-SSA, anti-SSB, anti-Sm, and anti-RNP were detected by using an immunoblotting technique. aCL and anti-β_2_-GPI were measured by enzyme-linked immunosorbent assay (ELISA) [23]. LA was measured in accordance with the recommendation of the Scientific and Standardization Committee of the International Society of Thrombosis and Hemostasis. Positivity for antiphospholipid antibodies (aPL) and LA was defined if confirmed in two separate measurements performed 12 weeks apart [24]. Healthy volunteers (*n* = 80) matched for age and sex ratio from the Ferrara University Hospital Blood Bank served as a control group.

### Sample collection and human lymphocyte preparation

Lymphocytes were isolated and prepared as previously described from the peripheral blood of control subjects and patients with SLE [25–27]. Leukocytes were separated from erythrocytes with a solution of 6 % dextran T500 (Sigma-Aldrich, St. Louis, MO, USA), suspended in Krebs-Ringer phosphate buffer, and layered onto 10 ml of Ficoll-Hypaque density gradient (GE Healthcare Life Sciences, Little Chalfont, UK).

After centrifugation, mononuclear cells were washed in 0.02 M phosphate-buffered saline (PBS) at pH 7.2 and containing 5 mM MgCl_2_ and 0.15 mM CaCl_2_. They were then decanted into a culture flask and placed in a humidified incubator (5 % CO_2_) for 2 h at 37 °C. This procedure, aimed at removing monocytes that adhered to the culture flasks, resulted in a purified lymphocyte preparation containing at least 99 % small lymphocytes identified by morphological criteria.

To obtain membrane suspensions, cell fractions were centrifuged in a hypotonic buffer at 20,000 × *g* for 10 minutes. The resulting pellet was incubated in Tris-HCl 50 mM buffer, pH 7.4, with 2 IU/ml adenosine deaminase (Sigma-Aldrich) for 30 minutes at 37 °C. After centrifugation at 40,000 × *g* for 10 minutes, the final pellet was used for radioligand binding assays. The protein concentration was determined by using a Bio-Rad Laboratories (Hercules, CA, USA) method with bovine serum albumin as the reference standard [25].

### Real-time reverse transcriptase-polymerase chain reaction experiments

Total cytoplasmic RNA was obtained from human lymphocytes by using the acid guanidinium thiocyanate phenol method. Quantitative real-time reverse transcriptase-polymerase chain reaction (RT-PCR) assay [25–28] of A_1_, A_2A_, A_2B_, and A_3_ ARs messenger RNAs (mRNAs) was performed using gene-specific fluorescently labeled TaqMan MGB Probe (minor groove binder) in an ABI Prism 7700 Sequence Detection System (Applied Biosystems, Foster City, CA, USA). Real-time RT-PCR for A_1_, A_2A_, A_2B_, and A_3_ ARs was carried out with the Assays-on-Demand TM Gene expression Products NM_000674, NM_000675, NM_000676, and NM_000677 (Applied Biosystems), respectively. For the real-time RT-PCR of the reference gene, the endogenous control human β-actin was used, and the probe was fluorescently labeled with VIC™ dye (Applied Biosystems).

### Western blot analysis

Human lymphocytes were washed with ice-cold PBS and lysed in radioimmunoprecipitation assay buffer (Sigma-Aldrich) containing protease inhibitors and 1 mM sodium orthovanadate. Proteins were eluted in Laemmli buffer, resolved by sodium dodecyl sulfate-polyacrylamide gel electrophoresis, and transferred to polyvinylidene fluoride membranes. Next, the membranes were incubated with specific antibodies for ARs (Alpha Diagnostic International, San Antonio, TX, USA), followed by washing and incubation with HRP-conjugated secondary antibodies. After a stripping step, the blots were reprobed with anti-β-actin antibody (clone EPR1123Y; EMD Millipore, Billerica, MA, USA).

### Saturation binding to ARs

Because AR mRNA and protein expression experiments in patients with SLE have shown an upregulation of A_2A_ARs compared with control subjects, we carried out experiments to examine saturation binding to this receptor subtype. For these assays, different concentrations of ^3^H-ZM 241385 (0.01–30 nM) as a radioligand, and cell membranes (60 μg of protein per assay) were incubated for 60 minutes at 4 °C [27]. The radioligand ^3^H-4-(2-(7-amino-2-(2-furyl)-[1, 2, 4]triazolo[2,3-a] [1, 3, 5] triazin-5-ylamino)ethyl)phenol (specific activity 27 Ci/mmol) was purchased from BIOTREND Chemikalien (Cologne, Germany). Nonspecific binding was determined in the presence of 1 μM ^3^H-ZM 241385. Bound and free radioactivity were separated by filtering the assay mixture through Whatman GF/B glass fiber filters (GE Healthcare Life Sciences) by using a Brandel cell harvester (Brandel, Gaithersburg, MD, USA) [28]. The filter-bound radioactivity was counted in a 2810TR liquid scintillation counter (PerkinElmer, Waltham, MA, USA).

### Pro- and anti-inflammatory cytokines release in cultured lymphocytes

Isolated lymphocytes from healthy subjects or patients with SLE were suspended at a density of 10^6^ cells/ml in RPMI 1640 medium supplemented with 2 % fetal bovine serum (EuroClone, Pero, Italy) and seeded into 24-well plates. Lymphocytes were incubated for 24 h in the absence or in the presence of an A_2A_AR agonist, CGS-21680 (2-*p*-(2-carboxyethyl)phenethylamino-5′-N-ethylcarboxamidoadenosine; 100 nM and 1 μM). In some experiments, cells were treated with a selective A_2A_AR antagonist, SCH 442416 (2-(2-furanyl)-7-[3-(4-methoxyphenyl)propyl]-7*H*-pyrazolo[4,3-*e*] [1, 2, 4] triazolo[1,5-*c*]pyrimidin-5-amine; 1 μM), 15 minutes before the agonist CGS-21680 to verify the specific involvement of these receptors in cytokine release. CGS-21680 was obtained from Sigma-Aldrich and SCH 442416 was purchased from Tocris Bioscience (Bristol, UK). At the end of incubation, the cell suspension was collected and centrifuged at 1000 × *g* for 10 minutes at 4 °C. IFN-α, TNF-α, IL-2, IL-6, IL-1β, and IL-10 levels were determined using a specific quantitative sandwich ELISA kit (R&D Systems, Minneapolis, MN, USA) according to the manufacturer’s instructions [25].

### Nuclear factor kB activation in human cultured lymphocytes

Nuclear extracts from human cultured lymphocytes of the examined patients were obtained by using a nuclear extract kit (Active Motif, Carlsbad, CA, USA) following the manufacturer’s instructions. Nuclear factor (NF)-kB subunit p65 activation was evaluated in lymphocyte nuclear extracts by using the TransAM NF-kB kit (Active Motif). The primary antibody against NF-kB recognized an epitope in the subunits that is accessible only when it is activated and bound to its DNA target. The reaction was developed with streptavidin-HRP, and optical density was read by spectrophotometry at 450-nm wavelength [27].

### Data and statistical analysis

Using dissociation equilibrium constants for saturation binding, affinity or *K*_d_ values, and the maximum densities of specific binding sites, B_max_ values were calculated for a system of one- or two-binding-site populations by nonlinear curve fitting [28]. All experimental data are reported as mean ± SEM of independent experiments as indicated in the figure legends. Statistical analysis of the data was performed by using Student’s *t* test or one-way analysis of variance (ANOVA) followed by Dunnett’s test. The analysis was carried out using the GraphPad Prism 5.0 statistical software package (GraphPad Software, La Jolla, CA, USA), and differences were considered statistically significant with a *p* value less than 0.01.

## Results

### Clinical characteristics

A total of 80 patients with SLE (71 women and 9 men) with a mean ± SD age of 44 ± 11.9 years, disease duration of 139 ± 100 months, and SLEDAI-2 K score of 4 ± 4.3 were studied. In addition, 80 healthy subjects matched for age and sex ratio were enrolled. Demographic, clinical, and pharmacological treatments of the study subjects are reported in Table 1.

**Table 1:** Clinical and demographic features of the study subjects, as well as pharmacological treatments in patients with systemic lupus erythematosus

	Patients with SLE (*n* = 80), *n* (%)
Clinical parameters	
Female/male	71/9
Age, years, mean ± SD	44 ± 11.9
Disease duration, months^a^	139 ± 100
SLEDAI-2 K score, mean ± SD	4 ± 4.3
SDI, mean ± SD	0.8 ± 1.2
Disease activity patterns	
CQD	46 (57.5 %)
CAD	18 (22.5 %)
MDA	14 (17.5 %)
RRD	2 (2.5 %)
Serological parameters	
aPL (aCL, β_2_-GPI, and/or LA)	39 (48.75 %)
ENA	48 (60 %)
Hypocomplementemia	53 (62.2 %)
Anti-dsDNA antibody, ongoing/previous	41 (51.2 %)/17 (21.1 %)
Treatments	
Corticosteroids, 2.5 up to 12.5 mg/day	67 (83.7 %)
Hydroxychloroquine, 200 mg/day	48 (60 %)
Ongoing immunosuppressant therapy	25 (31.2 %)
Mycophenolate mofetil	11 (13.7 %)
Cyclosporine A	4 (5 %)
Azathioprine	5 (6.2 %)
Methotrexate, 10–15 mg/week	3 (3.7 %)
Thalidomide	1 (1.2 %)
IVIg	1 (1.2 %)
PEX	1 (1.2 %)
Anticoagulants	11 (13.7)
Antiaggregant	33 (41.25)

*Abbreviations: SLEDAI-2 K* Systemic Lupus Erythematosus Disease Activity Index 2000, *SDI* Systemic Lupus International Collaborating Clinics/American College of Rheumatology Damage Index, *CQD* clinical quiescent disease, *MDA* minimal disease activity, *CAD* chronic active disease, *RRD* relapsing-remitting disease, *aPL* antiphospholipid antibodies, *aCL* anticardiolipin antibodies, LA lupus anticoagulant, *β*_*2*_ *-GPI* β_2_-glycoprotein I antibodies, *ENA* extractable nuclear antigen antibodies, *PEX* plasma exchange, *IVIg* intravenous immunoglobulin, *anti-dsDNA* anti-double-stranded DNA

^a^Disease duration at the time of sample collection

### A_2A_AR mRNA and protein expression are upregulated in lymphocytes of patients with SLE

**Fig. 1:**
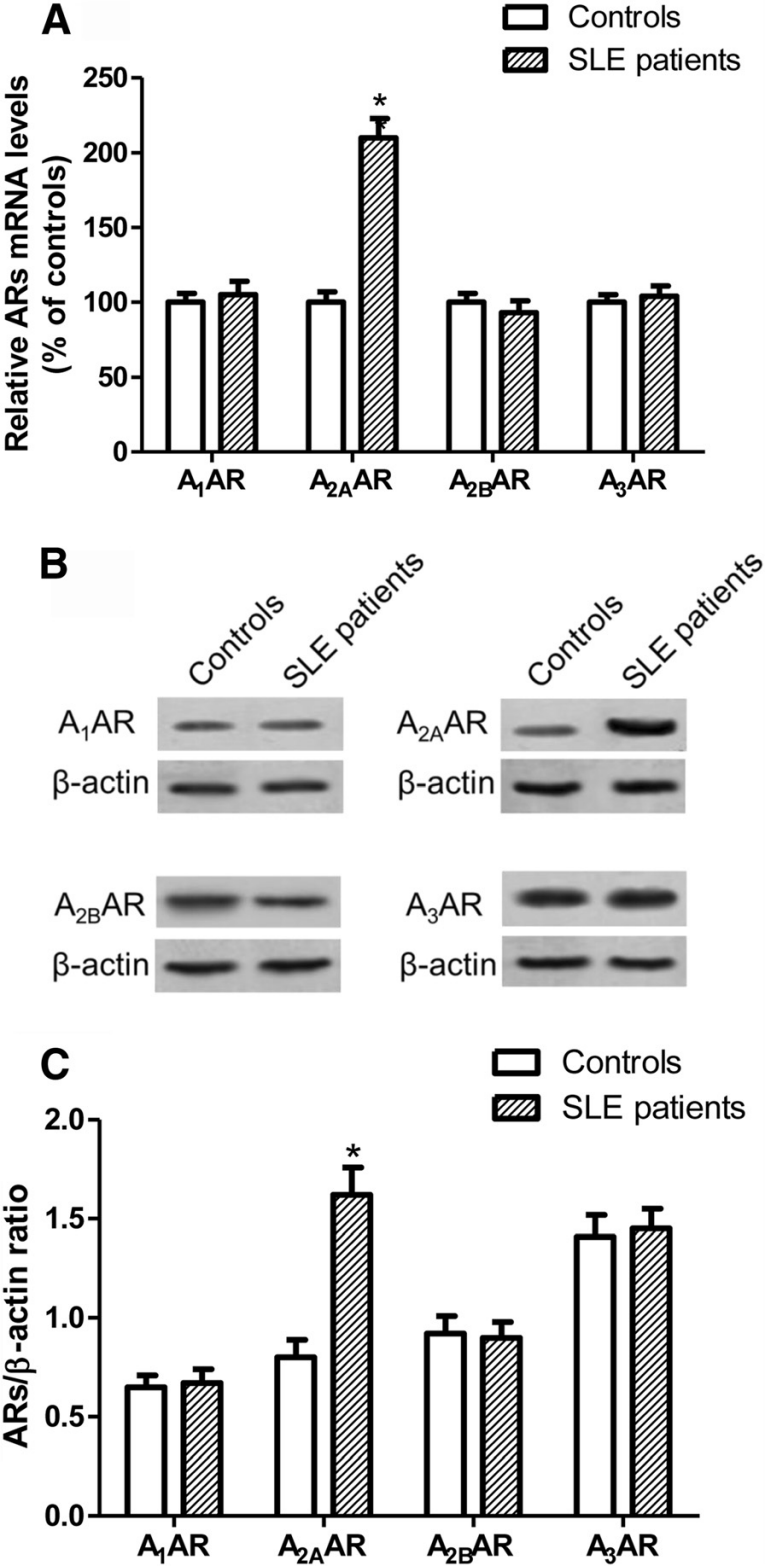
Messenger RNA (mRNA) and protein expression of adenosine receptors (ARs) in human lymphocytes from patients with systemic lupus erythematosus (SLE) and healthy subjects. **a** Relative AR mRNA levels were determined by real-time reverse transcriptase-polymerase chain reaction. Experiments were performed in duplicate with lymphocytes obtained from individual patients with SLE (*n* = 80) and healthy subjects (*n* = 80), and data are shown as mean ± SEM. **b** Western blot analysis showing immunoblot signals of ARs in one patient with SLE and one healthy subject, representative of blots obtained with lymphocytes from 80 patients with SLE and 80 healthy control subjects. β-actin was used as a loading control. **c** Densitometric analysis of AR expression in human lymphocytes from patients with SLE (*n* = 80) and healthy subjects (*n* = 80) indicated as a ratio of β-actin (loading control). Data are expressed as the mean ± SEM of densitometric analysis results obtained from the indicated number of subjects. * *p* < 0.01 versus control group by one-way analysis of variance with Dunnett’s test

AR mRNA and protein expression were evaluated in lymphocytes of patients with SLE in comparison with those of healthy subjects by means of quantitative RT-PCR assay and Western blot analysis, respectively. Figure 1a reports the relative A_1_, A_2A_, A_2B_, and A_3_ AR mRNA levels determined by RT-PCR in human lymphocytes of healthy subjects and patients with SLE. Among these receptors, only A_2A_AR mRNA expression was significantly increased in patients with SLE respect to control subjects. Western blot and densitometric analysis indicated a significant increase in A_2A_AR protein expression in lymphocytes of patients with SLE compared with those of healthy subjects, while no differences were found in A_1_, A_2B_, or A_3_ ARs (Fig. 1b, c).

### Alteration of A_2A_AR affinity and density in lymphocytes of patients with SLE

**Fig. 2:**
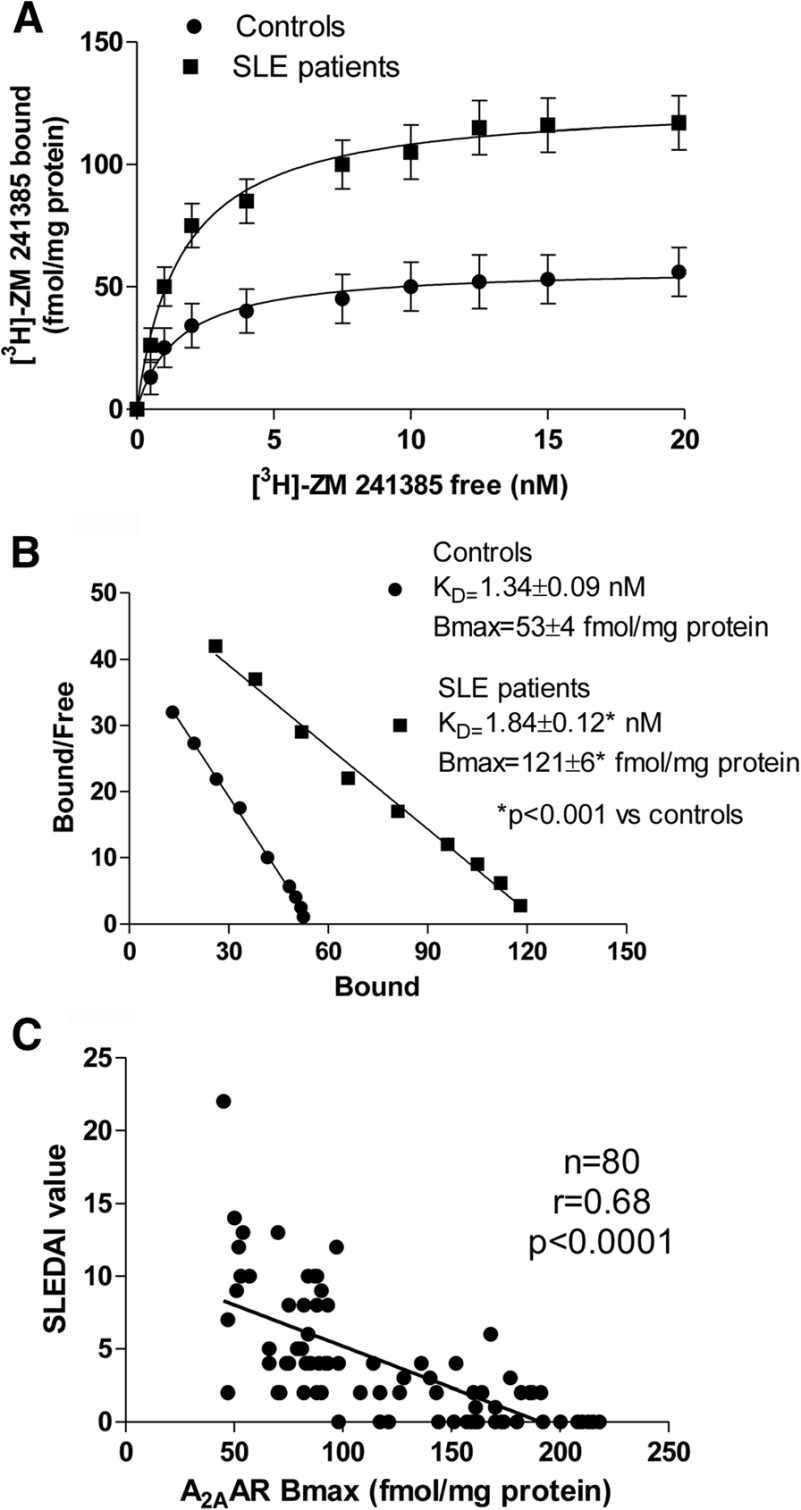
Adenosine 2A receptors (A_2A_ARs) are upregulated in lymphocytes from patients with systemic lupus erythematosus (SLE). **a** Saturation curves and **b** Scatchard plot showing the binding of ^3^H-ZM 241385 to A_2A_ARs in lymphocyte membranes derived from 80 healthy control subjects (solid circles) and 80 patients with SLE (solid squares) are also shown. Saturation binding experiments were performed as described in the Methods section. Data in the saturation curves are expressed as the mean ± SEM of results pooled from one experiment performed in duplicate for the indicated number of subjects. **c** Linear regression analysis between Systemic Lupus Erythematosus Disease Activity Index 2000 (SLEDAI-2 K) score and maximum number of A_2A_ARs (B_max_) in lymphocytes (*n* = 80, *r* = 0.68, *p* < 0.0001 by using Pearson’s or Spearman’s correlation (Pearson’s *r* = −0.68, Spearman’s *r* = −0.75)

Figure 2a and b show the saturation curves and Scatchard plots of [^3^H]-ZM 241385 in human lymphocytes, confirming the upregulation of A_2A_ARs in patients with SLE compared with healthy subjects. The affinity of the radioligand [^3^H]-ZM 241385 for A_2A_AR (expressed as *K*_d_, nanomoles) was decreased in lymphocytes from patients with SLE compared with that of the control group. Interestingly, the A_2A_AR density, expressed as a B_max_ value, significantly increased in patients with SLE compared with healthy subjects, reaching a 2.3-fold increment (Fig. 2a, b).

### Clinical correlations

An inverse correlation was found between A_2A_AR density (expressed as B_max_ values in femtomoles per milligram of protein), SLEDAI-2 K measured at the time of blood sampling (Fig. 2c), and disease activity through time evaluated according to the course patterns (CQD versus CAD; *p* < 0.0001). In addition, A_2A_AR density inversely correlated with serositis (*p* = 0.0043), hypocomplementemia (*p* = 0.0005), and anti-dsDNA positivity (Table 2). Regarding treatments, modulation of A_2A_AR density was found among corticosteroid users (*p* = 0.0078). With regard to A_2A_AR affinity, only one significant correlation was found with anti-dsDNA-positive patients (*p* = 0.008) (Table 2).

**Table 2:** Clinical, serological, and pharmacological treatments in patients with systemic lupus erythematosus: correlation with adenosine A_2A_ receptor affinity and density

	Patients (*n*)	*K*_d_ (nM)	*p* Value	B_max_ (fmol/mg of protein)	*p* Value
Disease activity patterns					
CQD/CAD	46/18	1.96 ± 1.19/1.77 ± 0.89	NS	141.6 ± 48.9/76 ± 28.6	< 0.0001
Organ involvement					
Renal, yes/no	13/67	1.33 ± 0.59/1.94 ± 1.15	0.06	114 ± 45.05/120.8 ± 52.3	NS
Neuropsychiatric, yes/no	20/60	1.73 ± 0.81/1.88 ± 1.18	NS	103.85 ± 48.2/125 ± 51.2	NS
Articular, yes/no	46/34	1.79 ± 1.22/1.9 ± 0.9	NS	110.85 ± 49.3/131.8 ± 51.5	0.06
Cutaneous, yes/no	25/55	1.46 ± 0.71/1.89 ± 1.2	NS	105.60 ± 43.9/123.8 ± 53.7	NS
Hematological, yes/no	41/39	1.70 ± 0.89/1.98 ± 1.28	NS	116.85 ± 47.8/123.6 ± 54.5	NS
Serositis, yes/no	21/59	1.60 ± 1.3/1.9 ± 0.9	NS	92.86 ± 38.7/129.2 ± 51.6	0.0043
Serological parameters					
aCL, yes/no	22/58	1.91 ± 1.01/1.81 ± 1.14	NS	134.04 ± 52.55/114.27 ± 47.3	NS
Anti-β_2_-GPI, yes/no	9/71	1.59 ± 0.46/1.87 ± 1.15	NS	116.11 ± 53.15/120.2 ± 51.1	NS
LA, yes/no	28/52	1.92 ± 1.20/1.8 ± 1.05	NS	109.03 ± 51.60/125.5 ± 50.2	NS
ENA, yes/no	58/22	1.92 ± 1.15/1.77 ± 1.07	NS	114,5 ± 47.34/123.1 ± 53.52	NS
Hypocomplementemia, yes/no	53/27	1.57 ± 0.78/2.15 ± 1.32	NS	97.88 ± 39.25/145.08 ± 51.78	< 0.0001
Anti-dsDNA, yes/no	41/39	1.65 ± 1.01/2.31 ± 1.18	0.008	106.26 ± 48.42/153.04 ± 41.76	< 0.0001
Treatments					
Corticosteroids, 2.5 up to 12.5 mg/day, yes/no	67/13	1.83 ± 1.17/1.88 ± 0.63	NS	113.12 ± 50.30/153.69 ± 41.47	0.0078
Hydroxychloroquine, 200 mg/day, yes/no	48/32	1.93 ± 1.07/1.69 ± 1.13	NS	126.89 ± 51.09/108.93 ± 49.72	NS
Immunosuppressants or induction therapy, yes/no	25/55	1.98 ± 1.19/1.54 ± 0.8	NS	124.31 ± 48.04/110.15 ± 56.46	NS
Anticoagulants, yes/no	11/69	2.01 ± 1.47/1.81 ± 1.04	NS	128.91 ± 64.01/128.91 ± 64	NS
Antiaggregants, yes/no	33/47	2.03 ± 1.24/1.7 ± 0.97	NS	123.39 ± 50.61117.13 ± 51.66	NS

*Abbreviations: aCL* anticardiolipin antibodies, *anti-β*_*2*_ *-GPI* anti-β_2_-glycoprotein I, *CAD* chronic active disease, *CQD* clinical quiescent disease, *dsDNA* double-stranded DNA, *ENA* extractable nuclear antigen antibodies, *LA* lupus anticoagulant, *NS* not significant

Analysis was carried out using unpaired *t* tests.

### A_2A_AR activation reduces proinflammatory cytokine production from lymphocytes

**Fig. 3:**
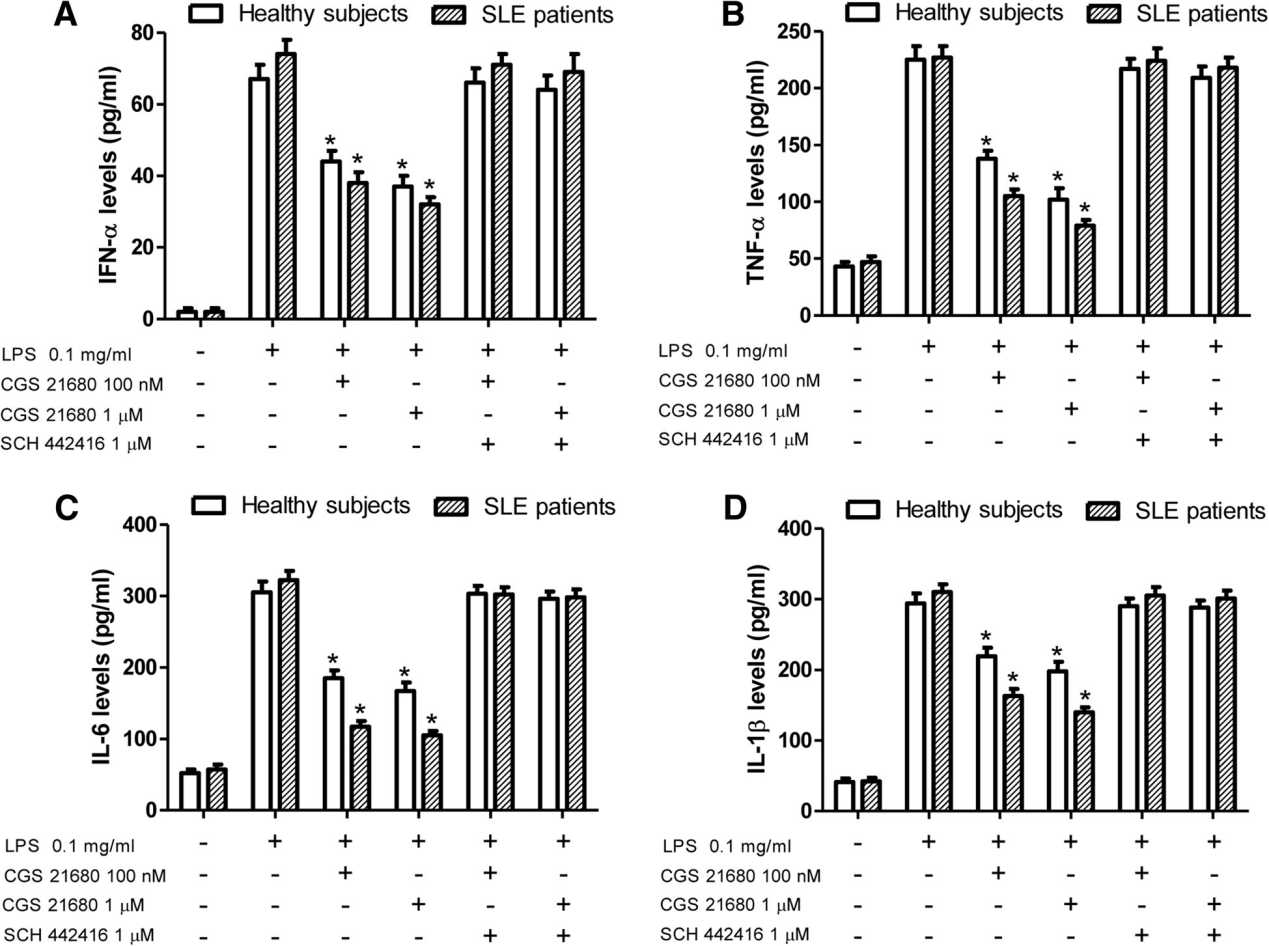
Adenosine 2A receptor (A_2A_AR) stimulation inhibits proinflammatory cytokine release. The effect of a well-known A_2A_AR agonist (CGS-21680; 100 nM and 1 μM) and antagonist (SCH 442416; 1 μM) on (**a**) interferon (IFN)-α, (**b**) tumor necrosis factor (TNF)-α, (**c**) interleukin (IL)-6, and (**d**) IL-1β release in cultured lymphocytes of patients with systemic lupus erythematosus (SLE) (*n* = 20) and healthy subjects (*n* = 20) that were stimulated by 0.1 mg/ml lipopolysaccharide (LPS) as determined by enzyme-linked immunosorbent assay. Data are expressed as the mean ± SEM of three independent experiments performed in triplicate * *p* < 0.01 versus LPS-treated cells by one-way analysis of variance with Dunnett’s test

**Fig. 4:**
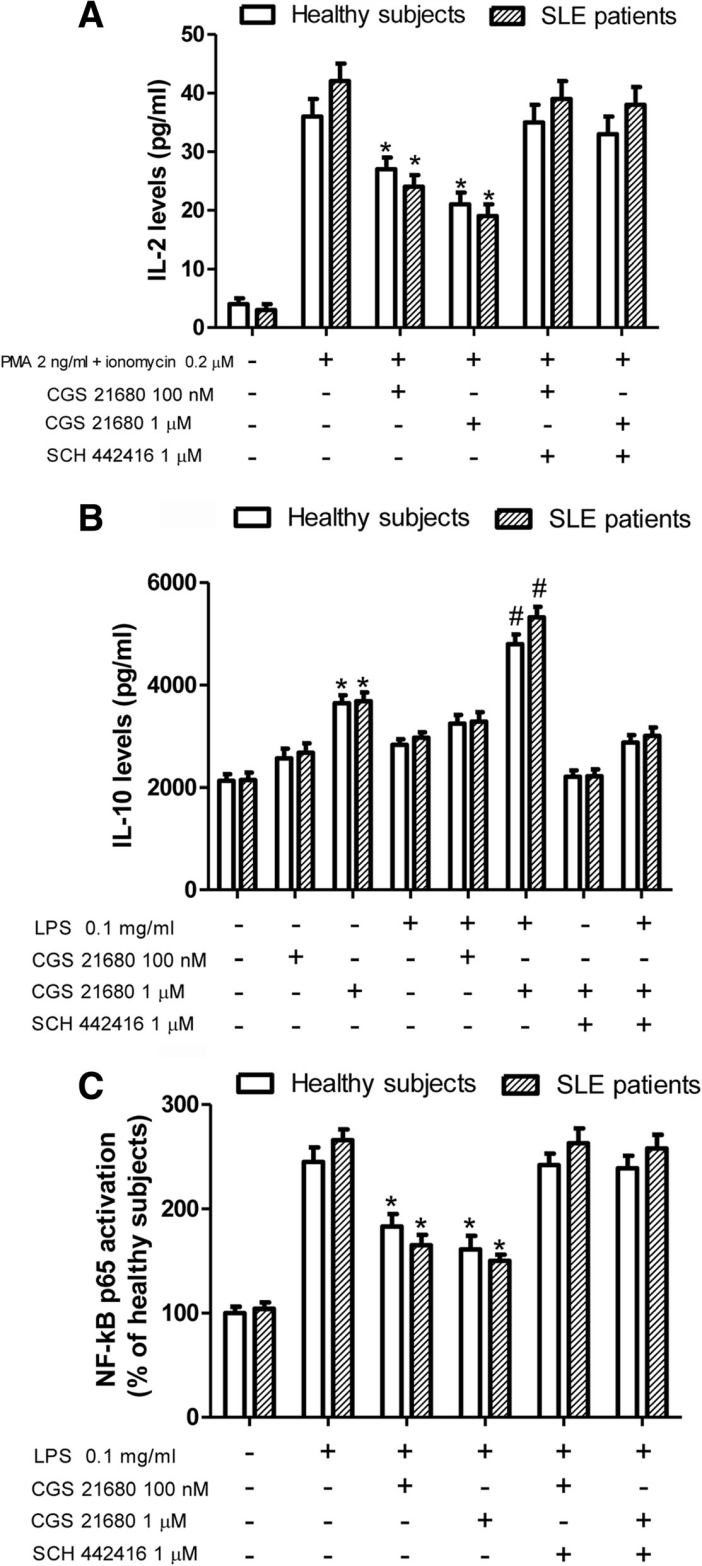
CGS-21680 effect on interleukin (IL)-2 and IL-10 release and on nuclear factor (NF)-kB activation. Effect of CGS-21680 (100 nM and 1 μM) and SCH 442416 (1 μM) in cultured lymphocytes of patients with systemic lupus erythematosus (SLE) (*n* = 20) and healthy subjects (*n* = 20) stimulated by lipopolysaccharide (LPS) (0.1 mg/ml) or phorbol 12-myristate 13-acetate (PMA) (2 ng/ml) and ionomycin (0.2 μM) on (**a**) IL-2 release, (**b**) IL-10 release, and (**c**) NF-kB activation. Data are expressed as the mean ± SEM of three independent experiments performed in triplicate * *p* < 0.01 versus LPS-treated cells by one-way analysis of variance with Dunnett’s test

To investigate the potential anti-inflammatory role of A_2A_AR stimulation in SLE, we evaluated the effect of CGS-21680 on the release of some of the most relevant proinflammatory cytokines involved in the pathogenesis of SLE, such as IFN-α, TNF-α, IL-6, IL-1β, and IL-2. In cultured lymphocytes of patients with SLE, a marked release of IFN-α was observed following the incubation of the cells with 0.1 mg/ml lipopolysaccharide (LPS) for 24 h (Fig. 3a). Interestingly, CGS-21680 at concentrations of 100 nM and 1 μM was able to inhibit the LPS-induced IFN-α release in lymphocytes of both patients with SLE and healthy subjects. However, the effect of CGS-21680 was significantly greater in lymphocytes obtained from patients with SLE than in those of healthy subjects (*p* < 0.0001), most likely due to the upregulation of A_2A_ARs (see Table 3). The inhibitory effect of the A_2A_AR agonist was counteracted by the selective antagonist SCH 442416 (1 μM), demonstrating the specific A_2A_AR-mediated response (Fig. 3a). Similar results were obtained when we evaluated the capability of CGS-21680 to inhibit the release of TNF-α induced by LPS (Fig. 3b). Again, the inhibitory effect of the A_2A_AR agonist was more evident in lymphocytes of patients with SLE than in those of healthy subjects (*p* < 0.0001) (see Table 3). The anti-inflammatory effect of A_2A_AR activation induced by CGS-21680 was also confirmed when we analyzed the production of other proinflammatory ILs, such as IL-6 (Fig. 3c), IL-1β (Fig. 3d), and IL-2 (Fig. 4a). As reported in Table 3, a greater inhibitory effect was obtained in lymphocytes of patients with SLE. Moreover, the use of the selective A_2A_AR antagonist SCH 442416 (1 μM) demonstrated that the effect was mediated by A_2A_ARs.

**Table 3:** Effect of CGS 21680 in lymphocytes from healthy subjects (*n* = 20) or SLE patients (*n* = 20) on different inflammatory mediators

	Healthy subjects			SLE patients		
	Cellular stimulation	CGS 21680 (1μM) stimulation	% of reduction/fold of increase	Cellular stimulation	CGS 21680 (1μM) stimulation	% of reduction/fold of increase
IFN-α	67 ± 4^a^	37 ± 3^a^	44.39 ± 1.21^d^	74 ± 4^a^	32 ± 2^a^	56.89 ± 1.52^d,^*
TNF-α	225 ± 12^a^	102 ± 10^a^	54.66 ± 1.14^d^	227 ± 10^a^	79 ± 5^a^	64.95 ± 2.33^d,^*
IL-6	305 ± 15^a^	167 ± 12^a^	45.38 ± 1.18^d^	322 ± 13^a^	105 ± 6^a^	67.71 ± 1.25^d,^*
IL-1β	294 ± 14^a^	198 ± 13^a^	32.64 ± 1.02^d^	310 ± 11^a^	140 ± 7^a^	54.20 ± 2.35^d,^*
IL-2	36 ± 3^b^	21 ± 2^b^	41.59 ± 1.08^d^	42 ± 3^b^	19 ± 2^b^	54.59 ± 1.76^d,^*
IL-10	2832 ± 112^a^	4802 ± 189^a^	1.70 ± 0.02^e^	2973 ± 104^a^	5329 ± 199^a^	1.79 ± 0.02^e,**^
NF-kB	245 ± 14^c^	161 ± 13^c^	34.07 ± 1.08^d^	266 ± 10^c^	150 ± 6^c^	43.69 ± 1.47^d,^*

The data are expressed as mean ± SEM; *p < 0.0001 vs healthy subjects; ^**^ *p* < 0.01 vs healthy subjects

^a^ Cellular stimulation with LPS; values in pg/ml

^b^ Cellular stimulation with PMA + ionomycin; values in pg/ml

^c^ Cellular stimulation with LPS; values in % of controls

^d^ Percentage of reduction

^e^ Fold of increase

### CGS-21680 increases production of anti-inflammatory cytokine IL-10 in lymphocytes

The incubation of lymphocytes with the A_2A_AR agonist CGS-21680 (1 μM) augmented basal IL-10 release in lymphocytes from healthy subjects and from patients with SLE (Fig. 4b). A more pronounced effect of CGS-21680 was obtained when cells were stimulated with LPS (0.1 mg/ml), although LPS alone did not alter IL-10 production. In the presence of LPS, the effect of CGS-21680 was significantly greater (*p* < 0.01) in lymphocytes of patients with SLE than in those of healthy subjects (see Table 3).

### A_2A_AR activation inhibits LPS-induced NF-kB activation in lymphocytes

Many of the anti-inflammatory effects of A_2A_AR stimulation are mediated by the inhibition of NF-kB activation [25]. To verify if CGS-21680 was able to inhibit NF-kB in lymphocytes from patients with SLE in comparison with those of healthy subjects, the activation of NF-kB p65 subunits following LPS treatment was investigated. As shown in Fig. 4c, the A_2A_AR agonist determined a marked reduction of LPS-stimulated NF-kB p65 subunit activation in nuclear extract from lymphocytes obtained from patients with SLE and healthy subjects, with a significantly greater effect in the former (see Table 3). The inhibitory effect of CGS-21680 was completely counteracted by the selective A_2A_AR antagonist SCH 442416 (1 μM).

## Discussion

The primary aim of this study was to investigate the role of ARs in SLE pathogenesis and to assess potential relationships between these receptors and clinical data. Within the complexity of the pathogenic mechanisms of lupus, innate immune responses play a significant role contributing either to tissue injury via release of inflammatory cytokines or to the aberrant hyperactivation of T and B cells, qualified as the most important players leading to autoreactive autoantibody production and resultant end-organ injury [3, 5, 29–31]. The role of the adenosinergic system is attractive for its multifunctionality in this wide spectrum of inflammation-related processes [10, 13, 14] and for its potential engagement in SLE.

AR mRNA and protein analysis supported higher A_2A_AR expression in lymphocytes from patients with SLE with than in those of control subjects, while no changes in A_1_, A_2B_, or A_3_ ARs were found, suggesting a specific A_2A_AR alteration. Moreover, saturation binding experiments confirmed the upregulation of A_2A_ARs in lymphocytes of patients with SLE.

Notably, the highest levels of A_2A_AR density were tightly correlated with the lowest levels of clinical—namely, clinimetric—indexes and serological parameters (anti-DNA, C3 and C4) of disease activity, suggesting that the endogenous activation of these receptors could lead to mitigation of the disease. This aspect is further supported by the finding of an inverse correlation between CAD progression of the disease and receptor density, suggesting that the mutual modulation of A_2A_AR expression identifies well a persistent and stable regulation of the inflammatory status.

The hypothesis that A_2A_AR upregulation could represent a compensatory mechanism to better counteract the inflammatory background in SLE is supported by a preclinical study in an MRL/lpr mouse model of lupus nephritis [32] in which the A_2A_AR mRNA expression in the kidneys of MRL/lpr mice was significantly increased compared with that in control mice. In this study, the treatment with an A_2A_AR agonist ameliorated the severity of nephritis and renal vasculitis and reduced leukocytic infiltration.

Because IFN-α plays a central role in SLE pathogenesis [5], we investigated the anti-inflammatory effect of A_2A_AR activation on this cytokine in cultured lymphocytes. The effects of the IFN signature on lupus lymphocytes have been studied mainly in the regulatory T-cell subpopulation, where the action of IFN-α diminished their activity [33], while in B cells it stimulated antibody production [34]. We demonstrated that the A_2A_AR agonist CGS-21680 inhibited IFN-α release in cultured lymphocytes with a greater effect in patients with SLE than in healthy subjects. This observation further supports the competence of A_2A_AR signaling, as suggested by a previous study [35], to promote peripheral tolerance by generation of regulatory T cells. The reduction of inflammatory response by A_2A_AR activation was also confirmed when we studied the release of typical proinflammatory cytokines such as TNF-α, IL-6, IL-1β, and IL-2. Furthermore, we found that CGS-21680 mediated a significant increase of anti-inflammatory IL-10, which is an important immunoregulator that supports T-cell differentiation and suppresses proinflammatory cytokines [36].

It is well known that activation of NF-kB pathways leads to enhanced B-cell survival and T-cell activation and maturation [37]. Moreover, NF-kB positively regulates gene-encoding cytokines and other inflammatory factors, suggesting that this transcription factor could be one of the master regulators of inflammatory responses. Thus, the inhibition of NF-kB by CGS-21680 could explain the reduction of LPS-stimulated proinflammatory cytokines in cultured lymphocytes of patients with SLE.

## Conclusions

Taken together, our data demonstrate the presence of A_2A_AR upregulation in patients with SLE and a significant inverse correlation of A_2A_AR density with SLEDAI-2 K score and CAD. The anti-inflammatory response of A_2A_ARs opens up a new perspective on the translational role of the A_2A_AR agonists in the pharmacological treatment of SLE, highlighting their therapeutic potential in the management of this disorder.

## Abbreviations

aCL: Anticardiolipin antibodies
ANA: Antinuclear antibody
ANOVA: Analysis of variance
anti-β_2_-GPI: Anti-β_2_-glycoprotein I
anti-RNP: Antiribonucloprotein
anti-SSA: Anti-Sjögren’s-syndrome-related antigen A
anti-SSB: Anti-Sjögren’s-syndrome-related antigen B
anti-Sm: anti-Smith
aPL: Antiphospholipid antibodies
AR: Adenosine receptor
C3 and C4: Complement components C3 and C4
CAD: Chronic active disease
CQD: Clinical quiescent disease
DC: Dendritic cell
dsDNA: Double-stranded DNA
ELISA: Enzyme-linked immunosorbent assay
ENA: Extractable nuclear antigen antibodies
IC: Immune complex
IFN-α: Interferon-α
IL: Interleukin
IVIg: Intravenous immunoglobulin
LA: Lupus anticoagulant
LPS: Lipopolysaccharide
MDA: Minimal disease activity
mRNA: Messenger RNA
NF-kB: Nuclear factor kB
NS: not significant
PBS: Phosphate-buffered saline
PEX: Plasma exchange
PMA: phorbol 12-myristate 13-acetate
RRD: Relapsing-remitting disease
RT-PCR: Reverse transcriptase-polymerase chain reaction
SDI: Systemic Lupus International Collaborating Clinics/American College of Rheumatology Damage Index
SLE: Systemic lupus erythematosus
SLEDAI-2 K: Systemic Lupus Erythematosus Disease Activity Index 2000
TNF-α: tumor necrosis factor-α
